# Preliminary Effects of a Multicomponent Exercise Program Delivered in Conventional and Snoezelen Rooms Among Older Women Attending Social Centers: A Non-Randomized Pilot Study

**DOI:** 10.3390/jfmk11030358

**Published:** 2026-09-08

**Authors:** Maria Beijinha, Miguel Jacinto, Rui Matos, Nuno Amaro, Diogo Monteiro, Nuno Couto, Raúl Antunes

**Affiliations:** 1 University of Leiria and Oeste, 2410-191 Leiria, Portugal; mariajoaobeijinha@hotmail.com (M.B.); miguel.s.jacinto@ulo.pt (M.J.); rui.matos@ulo.pt (R.M.); nuno.amaro@ulo.pt (N.A.); raul.antunes@ulo.pt (R.A.); 2 Research Center in Sport Sciences, Health Sciences and Human Development (CIDESD), 2410-191 Leiria, Portugal; 3 Sport Sciences School of Rio Maior, Santarém Polytechnic University, 2040-413 Rio Maior, Portugal; ncouto@esdrm.ipsantarem.pt; 4 Research Center in Sport Sciences, Health Sciences and Human Development (CIDESD), 2040-413 Rio Maior, Portugal

**Keywords:** exercise for older adults, Snoezelen, multisensory environment, active aging, quality of life

## Abstract

**Background**: The aging population in Portugal requires interventions such as multicomponent exercise to maintain the functionality of older adults. The aim of this exploratory pilot study was to investigate preliminary changes in body composition, physical fitness, quality of life and satisfaction with life following a low-frequency multicomponent exercise program delivered in two different community-based contexts (conventional exercise room and Snoezelen room). **Methods**: This study followed a non-randomized quasi-experimental methodology based on participant preference and logistical availability. Following the baseline assessment, participants self-selected their participation context. Participants who chose to participate in the exercise intervention were assigned to either the Snoezelen Room Group or the Exercise Room Group according to their preference, whereas participants who did not participate in either intervention because of personal preference or logistical constraints formed a non-participating comparison Control Group. The final sample comprised 27 older women: Snoezelen Room Group (*n* = 14), Exercise Room Group (*n* = 7), and Control Group (*n* = 6). The intervention consisted of a 12-week multicomponent program (1 session/week, 45–60 min) carried out in different environments. Body composition, functional physical fitness, quality of life and satisfaction with life were assessed before and after the intervention. **Results**: The primary between-group analysis of change scores (Δ = post−pre) identified significant differences for body mass (H(2) = 9.516, *p* = 0.009), with a significant Bonferroni contrast between the Snoezelen and Exercise Room Groups (1 vs. 2; adjusted *p* = 0.008). For Back Scratch, the Δ-based omnibus test was also significant (H(2) = 7.398, *p* = 0.025), with Snoezelen differing from the Exercise Room Group (1 vs. 2; adjusted *p* = 0.020). For quality of life, the Δ-based omnibus test was significant (H(2) = 11.356, *p* = 0.003), with significant contrasts between Exercise Room and Control Groups (2 vs. 3; adjusted *p* = 0.003). Secondary between-group analyses at specific time points also identified differences in post-intervention bone mass (H(2) = 8.460, *p* = 0.015; 1 vs. 2, adjusted *p* = 0.026; 2 vs. 3, adjusted *p* = 0.038), muscle mass (H(2) = 7.748, *p* = 0.021; 1 vs. 2, adjusted *p* = 0.034), Back Scratch (H(2) = 7.947, *p* = 0.031; 1 vs. 2, adjusted *p* = 0.031; 2 vs. 3, adjusted *p* = 0.050), and 6-Minute Walk (H(2) = 9.015, *p* = 0.011; 1 vs. 2, adjusted *p* = 0.012). **Conclusions**: The findings suggest that a low-frequency multicomponent exercise program may be feasible in community social centers; however, the observed within-group changes and exploratory between-group differences should be interpreted cautiously given the non-randomized design, small sample size, and baseline differences. Larger controlled studies are needed to determine whether the exercise environment contributes to differential outcomes.

## 1. Introduction

Population aging is a global demographic phenomenon with profound impacts on the health, well-being, and quality of life of older adults. In Portugal, this trend is particularly marked: 24.1% of the population is aged 65 or over, placing the country among the most aged in the European Union [1,2]. Globally, the proportion of people aged 60 or over is expected to rise from 12% to 22% by 2050 [3]. This rapid aging raises social, economic, and public health challenges, requiring interventions adapted to the functional and psychosocial needs of older populations [4].

Human aging is associated with a set of physiological and biopsychosocial changes that progressively compromise functionality. Among these changes, sarcopenia, decreased muscle strength and power, reduced cardiorespiratory performance, and declining balance and mobility stand out as central factors for maintaining physical capacity [5,6]. Studies indicate that muscle mass loss begins around the age of 30, accelerating after the age of 50, with estimated annual declines of 1–2% in muscle mass and 1.5–5% in strength [6]. These physiological declines contribute to an increased risk of falls, frailty, chronic disease, and functional disability, compromising autonomy and social participation. In addition to the physical impacts, these changes have direct repercussions on social involvement, subjective perception of health, and quality of life [7], and may negatively influence perceptions of well-being and satisfaction with life [8], reinforcing the need for an integrated assessment of functional and psychosocial aspects [9].

Quality of life is defined by the World Health Organization [10] as a multidimensional construct comprising several domains: physical, psychological, social and environmental, representing objective and subjective factors of well-being. Satisfaction with life reflects an individual’s cognitive evaluation of their own lives [11]. Both dimensions reveal a strong association with indicators of functional capacity, physical fitness, and autonomy [12]. The literature shows that adequate levels of strength, mobility, balance, and cardiorespiratory capacity not only reduce the risk of dependence and institutionalization but also reinforce more positive perceptions of health, well-being, and quality of life [12]. Recent systematic reviews have shown that regular physical exercise has moderate and consistent effects on improving quality of life, especially in the physical domain, through mechanisms such as improved functionality, self-perception of ability and social participation [13] and perception of satisfaction with life [14].

In this context, multicomponent exercise programs are particularly relevant from a clinical and functional perspective. These programs, which combine strength training, balance, mobility, walking and aerobic exercise, are highly effective in improving functional fitness, reducing the risk of falls and delaying frailty [15,16]. Cardiorespiratory capacity, especially VO_2_max, is one of the most important predictors of overall health, autonomy, and longevity [17]. Recent evidence shows that physically active older women, particularly those who practice aerobic activities, have better cardiac function, greater autonomy and lower levels of cardiovascular risk compared to their sedentary peers [17], reinforcing the role of exercise as a central intervention in healthy aging. This aspect is particularly relevant in older women, who have a higher prevalence of sarcopenia [18,19], a higher risk of frailty [20,21] and less involvement in formal exercise programs [22], justifying the specific focus on this population group.

In addition to approaches focused on physical condition, psychosocial and sensory interventions play a complementary role in the overall well-being of older adults. Among these, Snoezelen Multisensory Stimulation therapy stands out, initially developed for people with intellectual and developmental difficulties, but widely applied today in geriatric contexts [23]. A multisensory Snoezelen room consists of a controlled multisensory environment designed to stimulate the primary senses such as sight, hearing, touch, smell, and proprioception through equipment such as fiber optics, water columns, interactive lights, tactile surfaces, ambient music, aromas, and visual projections [24]. This type of intervention aims to promote relaxation, reduce anxiety, stimulate interaction and facilitate positive emotional responses. Studies show that Snoezelen intervention can improve mood, reduce agitation, enhance relaxation levels and promote social engagement in frail older adults and people with dementia [25,26]. More critically, two major gaps persist: first, most studies have focused on clinical populations (e.g., dementia), with a notable scarcity of experimental research evaluating Snoezelen’s applicability in healthy community-dwelling older adults; second, no study to date has systematically compared the effects of a multicomponent exercise program delivered in a Snoezelen room versus a conventional exercise room. These gaps limit the development of evidence-based recommendations for integrating multisensory environments into community exercise programs for healthy aging [27].

Considering the relevance of physical, sensory, and psychosocial dimensions in aging, it is crucial to understand in an integrated way how different types of intervention—from multicomponent exercise programs to multisensory approaches—can contribute to maintaining and promoting physical fitness, satisfaction with life, and quality of life in the older population. Research lacks studies that simultaneously compare these two types of intervention in real community settings, limiting the development of evidence-based practical recommendations. This integrated analysis is essential in a demographic context where population aging is intensifying and requires multidimensional responses aimed at promoting more active, functional and satisfying aging.

### Present Study

The literature indicates that multicomponent physical exercise, including strength exercise, balance and cardiorespiratory fitness, is an effective intervention for preserving functional capacity, preventing frailty and promoting autonomy in older adults, with potential positive effects on quality of life and satisfaction with life [15,28]. However, gaps remain in knowledge about the practical implementation of these programs in community settings, such as social centers, as well as their effectiveness [29,30]. Specifically, it is unclear: (i) whether low-frequency programs (e.g., one session per week) are sufficient to produce meaningful improvements in body composition and physical fitness; (ii) how the exercise environment (conventional vs. enriched sensory context) influences adherence, psychosocial responses, and functional outcomes; and (iii) whether the potential benefits of multisensory stimulation can be integrated with structured exercise to enhance quality of life and satisfaction with life approaches, such as Snoezelen therapy, have shown potential to stimulate sensory interaction, reduce anxiety, improve mood, and promote emotional well-being, but evidence regarding their application in healthy aging contexts is still limited and lacks rigorous experimental studies [24]. Unlike a conventional room, the Snoezelen environment reduces anxiety and the sensation of exertion through multisensory stimulation [31]. A lower perceived exertion allows older adults to exercise for longer or at a higher intensity, thereby increasing the effective dose of exercise [32]. Reduced stress and cortisol levels may also promote fat loss and muscle recovery [33]. However, these are hypotheses that need to be confirmed in future studies. Furthermore, a pleasant environment increases enjoyment, which predicts long-term adherence to physical activity [34]. Sensory stimuli (e.g., visual targets, tactile surfaces) can further improve body awareness and movement quality [27]. Thus, although the exercise programmed is identical, the Snoezelen context may represent a contextual factor potentially associated with different psychosocial responses during exercise. These mechanisms remain hypothetical and were not directly tested in this study. To the best of our knowledge, no studies have been identified that systematically compare the effectiveness of a multicomponent exercise program carried out in a conventional exercise room versus a Snoezelen room, simultaneously analyzing physical fitness, quality of life and satisfaction with life in older women [30].

Thus, this exploratory pilot study aimed to explore preliminary changes in body composition, physical fitness, quality of life and satisfaction with life following a multicomponent exercise program, applied in two different contexts—a conventional exercise room and a Snoezelen room—in older women who attend social centers. The aim is to investigate in an integrated manner the impact of these interventions on body composition, physical fitness, quality of life and satisfaction with life, examining not only functional gains but also changes associated with well-being and quality of life. Based on the existing literature, we formulated the following hypotheses: (H1) both intervention groups might demonstrate preliminary favorable trends in physical fitness, body composition quality of life and satisfaction with life [15,16,28]. Potential differences between exercise contexts were considered exploratory and hypothesis-generating.

## 2. Materials and Methods

### 2.1. Participants

This study was designed as an exploratory non-randomized pilot study intended to generate preliminary evidence and inform future randomized controlled trials. Participants were recruited at social centers in Óbidos, Portugal. To be eligible for the study, participants had to meet all of the following criteria: (i) aged 65 or over; (ii) regularly attend one of the social centers of the Óbidos Municipal Council, Portugal; (iii) independent mobility, i.e., able to walk without physical support from others; (iv) sufficient functional autonomy to perform the proposed exercises; (v) adequate understanding of the Portuguese language, allowing them to follow instructions and participate in assessments; (vi) any gender, race or ethnicity. Exclusion criteria were based on the ACSM contraindications for exercise testing and prescription [35]: (i) uncontrolled cardiovascular conditions (e.g., unstable angina, uncontrolled hypertension >180/110 mmHg, recent myocardial infarction <6 months); (ii) severe musculoskeletal limitations (e.g., recent fracture, uncontrolled arthritis, awaiting joint replacement) that would prevent safe performance of the exercises; (iii) uncontrolled metabolic conditions (e.g., uncontrolled diabetes with frequent hypoglycemic episodes); (iv) severe cognitive impairment (e.g., inability to understand simple instructions or follow safety guidelines); (v) acute illness or fever; or (vi) any other medical condition deemed by a physician to make physical exercise unsafe.

A power analysis (calculated using G*Power, version 3.1.9.7 [36,37]) showed that a sample of at least 18 participants in total was required to detect a medium effect size (ES) (α = 0.05, 1 − β = 0.95) using a repeated measures analysis of variance (ANOVA). An effect size (f) of 0.5 was chosen as this value has been reported in studies investigating the effects of exercise on the variables of interest in our study [38,39] (namely physical fitness and quality of life). The decision to use the ANOVA test is due to the fact that the software does not directly provide equivalent non-parametric tests [40,41], and its use is considered an acceptable and conservative approximation. Furthermore, evidence suggests that tests such as the Kruskal–Wallis test exhibit efficiency comparable to ANOVA under various conditions, particularly when the distributions of the groups are similar [40,41]. Thus, the approach adopted is consistent with methodological recommendations for studies in which the final analysis relies on non-parametric tests. However, the post hoc achieved power for between-group comparisons with the obtained sample (N = 27, unevenly distributed) was substantially lower, particularly for small-to-medium effects. Therefore, the study is best characterized as exploratory/pilot in nature, and results should be interpreted as hypothesis-generating rather than confirmatory.

### 2.2. Intervention

A multicomponent exercise program plan was implemented, focusing on physical fitness development, over 12 weeks, with one session per week per group, lasting between 45 and 60 min, carried out in the morning.

A shared multicomponent exercise framework was delivered in both intervention conditions. In the Exercise Room Group, the exercises were performed in a conventional space without additional sensory stimulation elements. In the Snoezelen Room Group, the same core exercise prescription (exercises, sets, repetitions, rest periods, and target intensity) was maintained, but the movements were delivered within the multisensory environment. Sensory adaptations were integrated into the context of the exercises—for example, participants performed balance exercises beside water columns, stretching exercises with light stimulation, and strength exercises while watching relaxing videos or feeling tactile stimuli—but these adaptations did not alter the prescribed motor task, target intensity, or rest periods. The sensory stimuli were contextual enhancements designed to enrich the exercise experience, not modifications to the exercise dose (see Appendix A for visual comparison. In the Snoezelen room, the movements were adapted to integrate the available sensory stimuli (e.g., balancing next to water columns, stretching with light stimulation), maintaining the muscle groups and intensity, while in the Exercise Room Group, they were performed in a conventional space, without additional stimulation elements. The sensory adaptations did not alter the motor task, intensity or rest periods. The exercise plan implemented in the sessions was developed based on the FITT-VP principles [35], promoting a gradual progression of the volume and intensity of the exercises according to the older adult group.

Each session consisted of activities structured in five blocks, in accordance with American College of Sports Medicine [35] recommendations, including cardiorespiratory exercises, upper and lower limb strength exercise, balance and flexibility exercises. The selection of exercises considered the functionality and safety of the participants, using simple materials such as dumbbells and free weights, balls, elastic bands, chairs and even a Pilates ring.

The exercise plan consisted of six distinct exercise sessions, organized to be repeated in two-week cycles, i.e., each exercise session was carried out for two consecutive weeks, allowing for better assimilation of the movements and progressive adaptation by the participants. In the second session of each cycle, a progression of exercise stimuli was introduced, which could take different forms: an increase in the duration of each exercise; an increase in the number of sets; an increase in the load used; in some cases, all these variables were adjusted simultaneously.

Over the 12 weeks, cardiorespiratory exercise consisted of walks with gradual progression in duration, intensity, and complexity. In the first four weeks, participants walked for 15 min at a moderate pace, increasing the incline during the walk. Between weeks 5 and 6, the duration increased to 20 min of continuous walking, maintaining a moderate pace and focusing on respiratory control, and maintaining a continuous pace was reinforced. Finally, in weeks 9 to 12, the walk reached 30 min, with an emphasis on a constant pace and encouragement of individual progression. Intensity was controlled using the Borg scale [42] and the Talk Test [43]. Exercise intensity was prescribed within a moderate perceived-exertion range (Borg 11–13). Borg ratings were recorded at the end of each exercise block (cardiorespiratory, strength, and balance). As no group-level summary of Borg ratings is presented in the current analysis, these data are not used to claim achieved equivalence of exercise intensity between conditions; rather, the two intervention conditions shared the same prescribed target intensity [42]. The Talk Test [43] was used as a supplementary measure: participants were able to speak in short sentences but not to sing. Intensity was recorded at the end of each exercise block (cardiorespiratory, strength, balance), with each participant asked to indicate their Borg scale rating. The values were noted on a session record sheet.

Strength exercises for both the upper and lower limbs began without weights, with simple movements. In weeks 1 and 2, participants performed 1 set of 10 to 15 repetitions of exercises such as squeezing a ball, squatting with chair support, leg curls with light weights, and holding their arms outstretched for 20 to 30 s. In weeks 3 and 4, new exercises were introduced, including vertically extended arms, shoulder rotation with dumbbells, step with support and heel raises, progressing to 2 sets of 8 repetitions, with the possibility of individual progression of load and time according to each participant’s tolerance. In weeks 5 and 6, strength was increased to 2 sets of 8 repetitions, incorporating bicep curls, horizontally extended arms, and lower limb exercises, allowing for gradual progression of the load according to each participant’s ability. Between weeks 7 and 8, the number of repetitions increased to 2 sets of 12 repetitions in more complete exercises. Finally, in weeks 9 to 12, the complexity of the exercises was increased, including shoulder presses, triceps with ball or kettlebell, abduction with elastic band and supine rowing, performed in 3 sets of 8 to 12 repetitions, with the possibility of increasing the load up to 2 kg depending on each participant’s tolerance (based on individual perceived exertion [42,43]).

Balance exercise began with simple tasks, such as walking in a straight line on a mark on the floor in the first few weeks. In weeks 3 and 4, moving around obstacles was introduced, gradually increasing the duration from 40 to 50 s. Between weeks 5 and 6, the exercises included walking while manipulating a moving balloon, promoting dynamic coordination. In weeks 7 and 8, unilateral balance was worked on, standing on one foot with one or both hands supported on the wall, increasing the duration from 50 to 60 s per leg. Finally, in weeks 9 to 12, the exercises began to include touching cones and hip extension in a lying position, promoting agility, postural control, and lateral movement.

All exercise sessions included stretching exercises at the end of the session, focusing on the arms and legs. In the initial weeks, each stretch was performed for 30 s per segment, increasing to 40 s in weeks 3 and 4. From week 5 to week 6, the duration increased to 50 s, and in weeks 7 to 12, the stretches reached 60 s per segment, promoting muscle relaxation and a gradual return to calm after the exercises.

The sessions were held with small groups, always with a maximum of 7 participants, respecting the space limitations of the Snoezelen room. The same rule was used for the group that carried out the practice in the Exercise Room Group in a normal context. The duration of each session ranged from 45 to 60 min.

### 2.3. Instruments

#### 2.3.1. Anthropometry

The first assessment carried out was anthropometry and body composition. The participants’ height was measured using a portable stadiometer (Topgim, Sintra, Portugal), and the values were recorded in centimeters (cm). Participants were asked to be barefoot for the measurement and to stand with their backs to the device, which was placed against the wall, with the back of their heads, backs and heels touching the wall and looking straight ahead. To take the measurement, the stadiometer was placed against the top of the head and the height was recorded.

#### 2.3.2. Body Composition

A bioimpedance scale (Tanita InnerScan V BC-601) was used to assess body composition, providing detailed information on body weight: body mass (kilograms), fat mass (percentage), bone mass (kilograms), total body water (percentage), muscle mass (kilograms) and visceral fat (index from 1 to 59). This methodology follows the recommendations of the American College of Sports Medicine [35]. Participants were given the following instructions prior to the assessment: (i) to avoid strenuous physical exercise in the 12 h preceding the assessment; (ii) to empty their bladder within 30 min of the measurement; (iv) to avoid consuming alcohol and caffeine in the 24 h prior to the assessment; and (v) to maintain their usual fluid intake on the day before the assessment. All assessments were carried out in the morning (between 8.00 am and 10.00 am), with participants barefoot and wearing as little clothing as possible. Participants were asked to be barefoot and to remove their coats and excess clothing (such as hats and scarves) and to keep only the most essential clothing on. Participants were then asked to step onto the scale, placing their feet on the reference marks with their weight evenly distributed across both feet, to look straight ahead and to remain still until asked to step off.

#### 2.3.3. Physical Fitness

To assess the various indicators of physical fitness, the Functional Physical Fitness Test Protocol from the Rikli and Jones Test Battery [44] was used, which aims to assess the physical components that are fundamental to functional autonomy, such as muscle strength, aerobic endurance, flexibility, agility, and balance. The Rikli and Jones Functional Fitness Test Battery shows high test–retest reliability (ICC = 0.80–0.98) and criterion validity for predicting functional independence in community-dwelling older adults [44].

To assess lower limb muscle endurance, the 30 s chair stand test was used [44]. The participant starts by sitting on a chair with their arms crossed over their chest and is instructed to perform as many repetitions of the movement of standing up and sitting down as possible for 30 consecutive seconds.

The 30 s Arm Curl Test was used to assess upper-limb muscle strength [44]. The standard protocol specifies a 2.27 kg dumbbell for women and 3.36 kg for men; in the present study, a 2 kg dumbbell was used for all female participants. In addition, both the right and left arms were assessed, whereas the standard test is generally performed using the dominant arm. Participants performed as many elbow flexion repetitions as possible during 30 s, with the arm starting in an extended position [44].

The Timed Get-Up-and-Go test was used to assess the participants’ agility, functional mobility, and speed and dynamic balance [44]. The procedure consists of the participant starting the test sitting on a chair, with their hands on their thighs and their feet flat on the floor. At the ‘go’ signal, the participant must get up from the chair as quickly as possible and walk 2.44 m as fast as possible around the cone without running, and sit down again. The time is stopped when the participant is completely seated again. Before the actual assessment, a trial was carried out to familiarize participants with the course and correct any errors in execution. The tests were then carried out, recording the time taken to complete the course.

The Chair Sit and Reach test was used to assess lower-limb flexibility [44]. The participant sat on the edge of a chair placed against a wall, with one leg extended and the heel on the floor and the other leg flexed. The participant slowly reached forward along the extended leg, keeping their hands together, and the maximal distance reached was recorded in centimeters using a ruler [44].

For the Back Scratch Test, upper-limb flexibility was assessed as the distance between the fingertips of the two hands when reaching behind the back [44]. The dominant hand was positioned over the same shoulder and reached downward, while the opposite hand reached upward from behind the back. The distance between the fingertips was measured in centimeters with a ruler. When the fingertips did not reach each other, the distance between them was recorded as a negative value; when the fingertips touched exactly, the score was recorded as 0 cm; and when the fingers overlapped, the overlap distance was recorded as a positive value. Thus, positive values represented overlap, values around zero represented exact contact, and negative values represented a remaining gap between the hands. Higher scores therefore indicated better upper-limb flexibility [44].

Finally, the 6-Minute Walk Test was performed to assess aerobic endurance [44]. The test aims to measure the maximum distance a participant could walk for six minutes along a 50-meter course, with 5-meter segments marked. To facilitate the process, a record sheet was used to count each lap completed by the participants. During the explanation of the test, the importance of participants maintaining a constant pace, without running, was emphasized, and they were encouraged to walk as far as possible, noting that they could slow down or stop whenever necessary, resuming walking as soon as possible. After the explanation, the participants went to the start of the course and, at the starting signal, began the test, which was measured with the aid of a stopwatch and cones to mark the course.

For all the tests, participants were encouraged to do their best.

#### 2.3.4. Quality of Life

The EUROHIS-QOL-8 was used, through the validated Portuguese version [45], in order to assess the quality of life of participants briefly and efficiently, measuring the overall perception of quality of life of each participant, encompassing different relevant dimensions, such as the physical and psychological health of each individual, as well as their social relationships and perception of financial life. The EUROHIS-QOL-8 has demonstrated good internal consistency (Cronbach’s α = 0.82–0.86) and convergent validity with the WHOQOL-Bref in older adult samples [45]. This instrument consists of eight items, developed from the generic WHOQOL-100 and WHOQOL-Bref instruments. Each of the domains (i.e., physical, psychological, social and environmental) is represented by two items. The result is an overall index corresponding to the sum of the eight items, in which a high value corresponds to a better perception of QoL. All response scales have a five-point format, ranging from ‘Not at all’ to ‘Completely’.

#### 2.3.5. Satisfaction with Life

The Satisfaction with Life Scale questionnaire, in its Portuguese version [46], validated for older adults by Antunes et al. [47], was used to assess an individual’s overall level of satisfaction with life. The Satisfaction with Life Scale (SWLS) shows acceptable internal consistency (Cronbach’s α = 0.78–0.89) and temporal stability (test–retest r = 0.82) in older populations [47,48]. This scale focuses only on the individual’s overall perception of their own life. It consists of five items, which are answered on a seven-point Likert scale, ranging from 1 (‘strongly disagree’) to 7 (‘strongly agree’). The items are then grouped into a single factor representing the overall satisfaction with life index (e.g., ‘In many areas, my life is close to my ideal’). The final score was calculated using the mean of the items.

### 2.4. Procedures

This study was submitted to the Ethics Committee of the Polytechnic Institute of Leiria, which issued opinion number CE/IPLEIRIA/102/2024.

The project had five main implementation phases. The first phase consisted of a meeting with local mayors to explain the objectives of the study and the implementation model, as well as the potential benefits/risks for the senior population. This was followed by a meeting with the respective social centers for the same purpose. After authorization for the implementation of the study, the project was presented to the older adults with a view to selecting those interested according to the defined criteria. They were provided with all the information relating to the project and informed consent to ensure that participation was voluntary and that all doubts had been clarified, as well as to inform them that they could withdraw from the study at any time and for any reason. After this, initial assessments of the participants were carried out, including physical tests and written tests. First, anthropometric and body composition assessments were performed on the participants, followed by physical tests and, finally, the instruments were applied. Following the baseline assessments, participants self-selected their participation context rather than being randomly allocated. Participants who wished to participate in the exercise intervention chose between the Exercise Room Group, in which the multicomponent exercise program was performed in a conventional exercise room, and the Snoezelen Room Group, in which the same exercise program was performed in a Snoezelen room. Participants who did not participate in either intervention because they preferred not to participate or were unable to participate for logistical reasons formed the non-participating comparison Control Group. Thus, group assignment was determined by participant preference and/or logistical non-participation and was not randomized. At the end of the 12-week intervention period, all participants who remained in the study underwent the same post-intervention assessments. Participants in the Control Group did not participate in any exercise sessions during the intervention period and completed both the baseline and post-intervention assessments. Finally, at the end of all the exercise sessions, all participants took all the tests again. The assessments were carried out in the spaces of each Center, so individuals who took the initial and final assessments and who never participated in the exercise sessions were included in the Control Group. These assessments were carried out by the investigator responsible for the study. At the end of each session, the participants’ satisfaction with the class was also assessed, which was an important factor for the management of the program. At the end of the sessions, participants were asked to vote by placing a token in one of the three boxes provided, each representing a different level of satisfaction with the session (green with a smile for a positive rating, yellow for neutral, and red for negative).

All sessions were planned and supervised by a physical exercise instructor who was qualified and certified for that purpose. The same qualified and certified physical exercise instructor supervised all exercise sessions in both intervention conditions.

### 2.5. Statistical Analysis

Descriptive statistics were calculated using means and standard deviations for all variables. Normality and homogeneity of variance were assessed using the Shapiro–Wilk and Levene tests, respectively.

The primary comparative analysis evaluated differences between groups in the magnitude of change from pre- to post-intervention. For each outcome, an individual change score was calculated as Δ = post-intervention − pre-intervention. Differences in Δ among the three groups (Snoezelen Room Group, Exercise Room Group, and Control Group) were examined using the Kruskal–Wallis H test, given the small sample sizes and the non-normal distribution observed for several variables.

When the omnibus Kruskal–Wallis test was statistically significant, pairwise post hoc comparisons were performed using the Bonferroni adjustment. The Bonferroni correction was applied within each family of three pairwise group comparisons for a given outcome, resulting in an adjusted significance. The *p*-values reported for these pairwise comparisons are Bonferroni-adjusted *p*-values [49].

The Wilcoxon signed-rank test was used as a supplementary exploratory analysis to assess pre–post changes within each group. For each Wilcoxon comparison, the effect size was calculated by dividing the standardized test statistic by the square root of the total number of paired observations, where N represents the number of paired observations included in that specific within-group comparison. Effect sizes were interpreted as small (r ≥ 0.10), medium (r ≥ 0.30), and large (r ≥ 0.50) [50,51].

The omnibus Kruskal–Wallis statistic is reported as H, whereas the statistic associated with pairwise post hoc comparisons is reported separately according to the post hoc procedure. The Wilcoxon signed-rank statistic is reported as Z.

All analyses were conducted using IBM SPSS Statistics, version 29 (IBM Corporation, Armonk, NY, USA), with a two-sided significance level of α = 0.05.

## 3. Results

**Figure 1:**
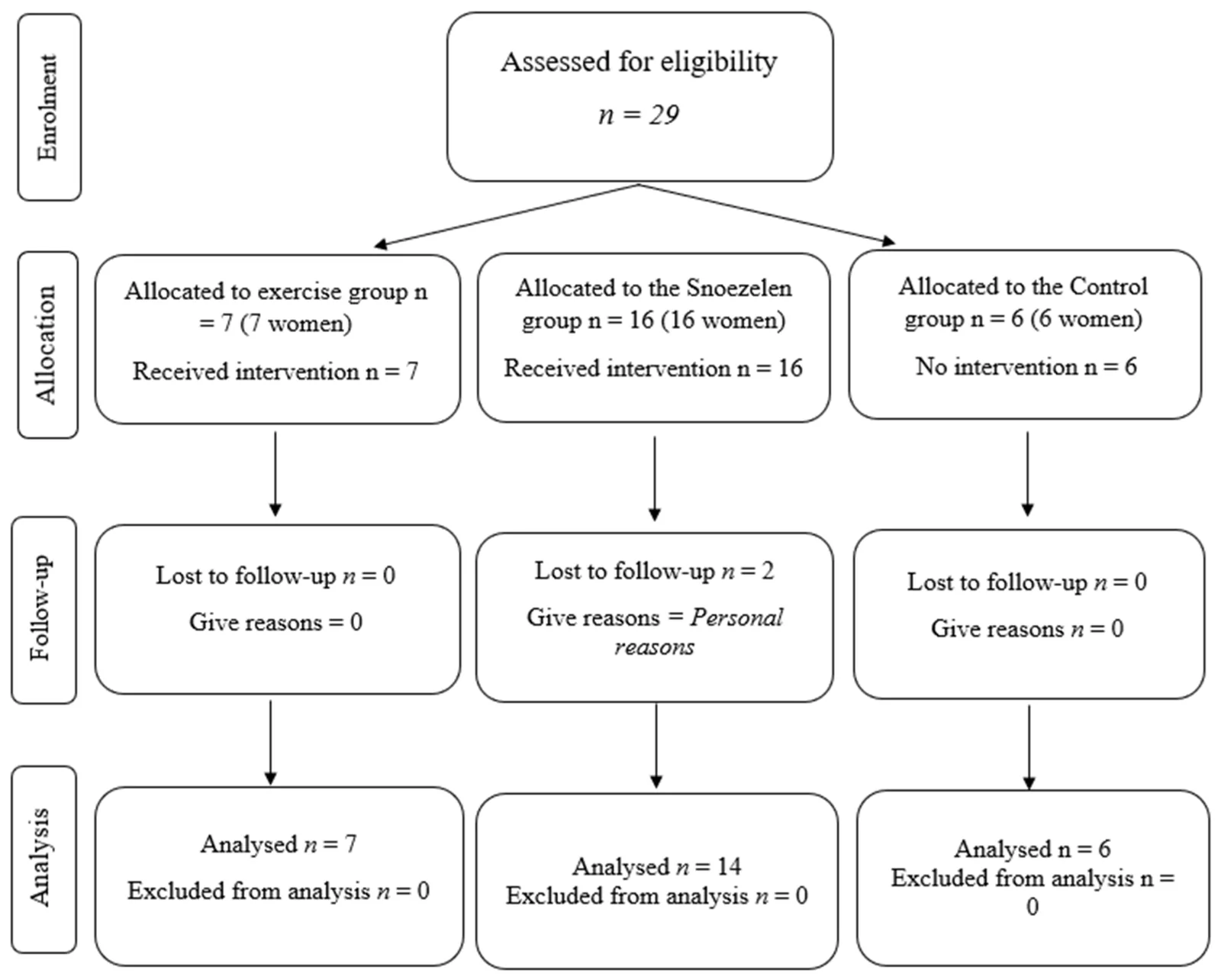
Flowchart of the groups under study.

The sample for this study consisted of 29 older women who attend social centers in Óbidos, Portugal, with a mean age of 78.96 years and a standard deviation of 7.87 years (Figure 1). The participants were aged between 65 and 95 years. After presenting the studies to eligible older adults, they were given the opportunity to decide whether they wanted to participate and in what context. Seven participants chose to participate in the group that carried out the intervention in a conventional exercise room, 16 chose to do so in the Snoezelen room, and six did not want to participate in the intervention groups or did not have time to do so, agreeing to be part of the Control Group. An attendance criterion of ≥75% of the scheduled exercise sessions was established as the minimum adherence threshold for the intervention groups. This criterion was not used to determine initial group allocation. No participant was excluded from the final analysis because of attendance below 75%. Two participants withdrew from the study for personal reasons unrelated to the exercise program; consequently, the final analytical sample comprised 27 participants. Among participants who completed the intervention, attendance was 95.23% in the Exercise Room Group and 90.47% in the Snoezelen Room Group for all sessions conducted.

Table 1 presents the descriptive statistics associated with the body composition variables of the participants in the three groups studied: Snoezelen Room Exercise Group, Exercise Room Group, and Control Group. The data is organized according to the two assessment moments (pre- and post-intervention), allowing us to observe the possible changes that occurred during the intervention period.

The descriptive statistics showed a decrease in body mass in both intervention groups and an increase in the Control Group. The primary Δ-based Kruskal–Wallis analysis identified a significant between-group difference in body mass, H(2) = 9.516, *p* = 0.009. Using the group coding 1 = Snoezelen, 2 = Exercise Room, and 3 = Control, Bonferroni testing showed a significant contrast only between groups 1 and 2 (post−pre change; H = −3.012, adjusted *p* = 0.008), with the Snoezelen Group showing the larger reduction in body mass. No other Δ-based body-composition outcome showed a significant between-group difference.

The descriptive statistics of the physical fitness test results for the three study groups at the two test assessment times (pre- and post-intervention) are described in Table 2.

The descriptive results showed improvements in several strength outcomes in both intervention groups, but the primary Δ-based analysis identified a significant between-group difference only for Back Scratch. For Back Scratch, the omnibus Kruskal–Wallis test was significant, H(2) = 7.398, *p* = 0.025, and Bonferroni testing showed a significant contrast between groups 1 and 2 (Snoezelen vs. Exercise Room; H = 2.719, adjusted *p* = 0.020). The positive Δ in the Snoezelen Group (+5.57 cm) contrasted with the negative Δ in the Exercise Room Group (−5.85 cm). No other Δ-based physical fitness outcome showed a significant between-group difference.

Table 3 presents the descriptive statistics of the results obtained through the Quality of Life Scale and the Satisfaction with Life Scale.

The descriptive results appear to indicate improvements in the perception of quality of life across all study groups. For the primary Δ-based analysis, a significant between-group difference was observed for quality of life, H(2) = 11.356, *p* = 0.003. Bonferroni testing identified a significant contrast between groups 2 and 3 (Exercise Room vs. Control; Post hoc statistic = 3.293, adjusted *p* = 0.003), indicating that the Exercise Room Group showed a significantly greater improvement in quality of life compared to the Control Group.

For satisfaction with life, the Δ-based omnibus test was not significant, H(2) = 4.452, *p* = 0.108, indicating no statistically detectable differences between the three groups in terms of change over time. Although the Snoezelen Group showed a numerically larger mean improvement (Δ = +0.45) compared to the Exercise Room Group (Δ = +0.02) and the Control Group (Δ = +0.26), these differences did not reach statistical significance.

Table 4 presents the results of the body composition analysis of the participants in the three study groups.

Most pre-intervention body-composition comparisons were not statistically significant. At post-intervention, significant omnibus differences were observed for bone mass, H(2) = 8.460, *p* = 0.015, and muscle mass, H(2) = 7.748, *p* = 0.021. For bone mass, Bonferroni testing showed differences between groups 1 and 2 (H = 2.630, adjusted *p* = 0.026) and groups 2 and 3 (H = −2.492, adjusted *p* = 0.038), with the Exercise Room Group showing lower post-intervention bone mass than the Snoezelen and Control Groups. For muscle mass, the significant contrast was between groups 1 and 2 (H = 2.527, adjusted *p* = 0.034), with higher post-intervention muscle mass in the Snoezelen Group. The primary Δ analysis showed a significant difference only for body mass, H(2) = 9.516, *p* = 0.009, with groups 1 and 2 differing (H = −3.012, adjusted *p* = 0.008). Supplementary within-group analyses indicated significant pre–post changes in several body-composition outcomes, but these do not constitute between-group evidence.

Significant changes were identified between moments, especially in the Snoezelen Group: (i) body mass: significant decrease in the Snoezelen Group, while the Control Group showed a significant increase; (ii) fat mass (%): significant reduction only in the Snoezelen Group; (iii) total body water (%): significant increase in the Snoezelen Group; (iv) visceral fat: significant decrease in the Snoezelen Group and also in the Exercise Room Group; (v) regarding bone mass and muscle mass, no significant differences were observed between moments.

Table 5 shows the assessment of the functional physical fitness of the participants in the three groups.

For functional fitness, the primary Δ-based analysis identified a significant between-group difference only for Back Scratch (H(2) = 7.398, *p* = 0.025; groups 1 vs. 2, H = 2.719, adjusted *p* = 0.020), with a more favorable change in the Snoezelen Group than in the Exercise Room Group. Secondary analyses at specific time points showed significant group differences for the 6-Minute Walk Test at baseline (H(2) = 13.096, *p* = 0.001; groups 1 vs. 2, H = −3.619, adjusted *p* = 0.001) and post-intervention (H(2) = 9.015, *p* = 0.011; groups 1 vs. 2, H = −2.871, adjusted *p* = 0.012), with higher values in the Exercise Room Group. At post-intervention, Back Scratch also differed between groups (H(2) = 7.947, *p* = 0.031; groups 1 vs. 2, H = 2.565, adjusted *p* = 0.031; groups 2 vs. 3, H = −2.396, adjusted *p* = 0.050), with the Exercise Room Group showing the lowest post-intervention value. No other primary Δ-based physical fitness outcome showed a significant between-group difference.

Table 6 shows the results of quality of life and satisfaction with life for the three groups in the study, before and after the intervention.

At baseline, no statistically significant between-group differences were observed for quality of life or satisfaction with life. At post-intervention, quality of life differed significantly between groups, H(2) = 12.659, *p* = 0.002, with significant contrasts between groups 1 and 2 (H = −2.676, adjusted *p* = 0.022) and groups 2 and 3 (H = 3.435, adjusted *p* = 0.002); the Exercise Room Group had the highest post-intervention quality of life score. Satisfaction with life also differed at post-intervention, H(2) = 9.037, *p* = 0.011, with a significant contrast between groups 1 and 2 (H = 2.883, adjusted *p* = 0.012), with the Snoezelen Group having the higher score.

Regarding the evolution over time and in terms of quality of life, there was a significant increase in the Snoezelen and Exercise Room Groups, with moderate effect sizes, while the Control Group showed no relevant changes. Regarding satisfaction with life, a significant improvement was observed only in the Snoezelen Group, with no significant changes in the other groups. The analysis of the variations revealed statistically significant differences between the groups in the Satisfaction with Life variable, indicating distinct patterns of change throughout the intervention.

The results show a high level of acceptance of the sessions in both groups, with most participants reporting that they enjoyed the activity. The Snoezelen Group (comprising participants from the Vau and Capaleira social centers) had a slightly higher percentage of “Liked it” responses (164/168 votes, 97.6%) compared to the Exercise Room Group (Gaeiras social center; 78/80 votes, 97.5%). “So-so” responses accounted for 2.4% (4/168) and 2.5% (2/80), respectively. No “Did not like” responses were recorded in either group, indicating a very positive overall perception of the interventions across both exercise environments.

## 4. Discussion

The aim of this study was to analyze and compare the effects of a multicomponent physical exercise program carried out in two different contexts—a conventional exercise room and a Snoezelen room—on body composition, functional physical fitness, quality of life and satisfaction with life in older women in the community. Exploratory within-group analyses showed pre–post changes in both intervention groups. These results support the hypothesis that the exercise environment may be associated with different adaptation profiles. The between-group comparisons of change scores (Δ) revealed few statistically significant differences between the two intervention groups. For most outcomes, the analysis of Δ did not show superiority of either environment. Regarding within-group pre–post analysis, the results appear to indicate that both intervention environments were associated with significant pre–post changes, although with different adaptation profiles, suggesting that the environment in which exercise is performed may be associated with differences in physical and psychosocial outcomes. The results obtained corroborate the literature that supports multicomponent exercise as a central intervention in promoting active aging, with positive impacts on functionality, metabolic health, and quality of life [52,53]. However, the results of this study (namely within-group pre–post analyses) add original evidence by demonstrating that the incorporation of a multisensory environment was accompanied by differences in certain outcomes, particularly in body composition and variables related to subjective well-being, even when the exercise structure was similar.

### 4.1. Body Composition

In the Snoezelen Group, we observed a considerable decrease in total body mass, the percentage of fat mass, and visceral fat, followed by an increase in the percentage of body water. On the other hand, the Exercise Room Group showed only a significant reduction in visceral fat, while the Control Group showed an increase in total body mass.

The pairwise comparison based on post–pre intervention differences (Δ) confirmed this more pronounced variation in the Snoezelen Group regarding body mass, which was associated with a change with intervention compared to its absence.

The results of several analyses provide preliminary indicators that a multisensory environment may be associated with favorable changes in body composition, particularly fat mass and body water [54]. However, due to the absence of baseline adjustment and the non-randomized design, these findings should be interpreted with caution. It is not possible to conclude that the Snoezelen environment is superior to the conventional exercise room for improving body composition. One possible explanation for these effects resides in the reduction in stress levels provided by the Snoezelen context, which is characterized by visual (e.g., images), auditory (e.g., music), and tactile (e.g., different textures) stimuli that induce a feeling of relaxation. Studies show that reduced stress levels and improved emotional regulation can positively affect the neuroendocrine parameters involved in energy use and fat distribution [31,33,55]. However, stress levels were not directly measured in this study, and this interpretation remains hypothetical. In addition, even though it was not monitored, participation in this context may have encouraged greater participation, greater involvement/spontaneous movement, and exercise tolerance, contributing to a greater effective dose of exercise (i.e., more attendance, fewer interruptions, and more intensity), which are crucial factors for changes in body composition [32].

On the other hand, the lack of significant variations in the remaining variables may be related to the short duration of the intervention program and the lower sensitivity of bioimpedance to detect small intergroup changes [56]. However, the literature indicates that varied exercise programs can generate modest but clinically relevant improvements in the body composition of older adults, even without changes in diet [57,58].

On the other hand, the Control Group showed negative results, such as total body mass gain. This finding is consistent with longitudinal studies showing that the absence of regular physical activity accelerates unfavorable changes in body composition in older age [6].

### 4.2. Functional Physical Fitness

Regarding functional fitness, the results show significant improvements in lower and upper limb strength in the Snoezelen and Exercise Room Groups, indicating the potential effectiveness of multicomponent exercise even with reduced weekly frequency. These results are in line with studies that demonstrate relevant functional gains in older adults with moderate exercise volumes, provided there is consistency and adequate progression [52,57]. Although the Snoezelen group showed large within-group effect sizes for lower limb strength, the between-group comparison of Δ did not reveal statistically significant differences between the two intervention groups [59]. The Exercise Room Group demonstrated significant improvements in both the right and left arms. The Snoezelen Group showed, in exploratory within-group pre–post analyses an increase in the right arm, with no significant change in the left arm. Despite these intra-group differences, the absence of inter-group differences in change scores does not provide evidence of a differential effect between intervention models.

Lower limb flexibility did not show significant changes in any of the groups, possibly due to the low volume of stimuli (despite specific exercises with movements similar to those in the assessment test being prescribed) and the short duration of the intervention [35,60]. On the other hand, upper limb flexibility improved significantly in the Snoezelen Group, in contrast to the Exercise Room Group. One possible explanation, which remains speculative because relaxation and muscle tone were not assessed in the present study, is that multisensory stimulation may influence perceived relaxation [31] and movement-related responses [26,27].

Regarding agility, functional mobility, and dynamic balance, assessed by the Timed Get-Up-and-Go Test, no significant inter- or intra-group variations or differences were observed, indicating that the intervention may not have been sufficiently specific, intense, or of adequate volume to promote improvements in this capacity.

Regarding cardiorespiratory capacity, only the Exercise Room Group showed significant improvements in the 6 min Walk Test, reflecting the greater specificity of the aerobic stimulus and possible baseline differences between the groups [32,60], differences that were evident in the pre-intervention assessment.

### 4.3. Quality of Life and Satisfaction with Life

Exploratory within-group pre–post analyses indicated increases in quality of life scores in both intervention groups, with the Exercise Room Group showing the largest mean improvement (Δ = +4.42), followed by the Snoezelen Group (Δ = +2.14). The between-group Δ analysis detected a statistically significant difference (H(2) = 11.356, *p* = 0.003), with Bonferroni post hoc testing revealing a significant contrast between the Exercise Room Group and the Control Group (Post hoc statistic = 3.293, adjusted *p* = 0.003). This suggests that participation in the exercise program, particularly in the conventional exercise room, was associated with greater improvements in quality of life compared to non-participation. Improvements in quality of life in older adults have been associated with improvements in the perception of functionality, autonomy, and social participation, dimensions that were addressed in both intervention contexts [12,13]. Thus, the improvements observed in quality of life are consistent with the functional gains recorded, although the larger improvement in the Exercise Room Group warrants further investigation.

Regarding satisfaction with life, the Snoezelen Group showed a numerically larger mean improvement (Δ = +0.45) compared to the Exercise Room Group (Δ = +0.02) and the Control Group (Δ = +0.26). However, the between-group Δ analysis did not detect a statistically significant difference (H(2) = 4.452, *p* = 0.108), indicating that the observed numerical differences between groups could be due to chance. Although within-group pre–post analyses suggested a significant increase in the Snoezelen group, these findings should be interpreted with caution given the non-significant between-group comparison. The observed pattern raises a hypothesis that the multisensory context may be relevant to subjective well-being outcomes; however, the present design does not allow an environmental effect to be established, particularly because the group differences at baseline and the non-randomized design limit causal inference. As described above, these multisensory environments have been associated with emotional regulation, reduction in anxiety, depression and stress symptoms, and improved mood, creating more positive experiences during practice [25]. However, due to baseline differences between groups and the non-randomized design, no conclusion of superiority of one environment over the other can be drawn. Future randomized controlled trials with baseline adjustment are needed to determine whether multisensory environments confer additional benefits beyond those of conventional exercise spaces.

### 4.4. Practical Implications and Limitations of the Study

The findings provide preliminary support for the feasibility of delivering a low-frequency multicomponent exercise program in community social centers. However, the small non-randomized sample and baseline differences mean that the observed changes should be considered exploratory and hypothesis-generating rather than evidence of intervention effectiveness. Future controlled studies should examine whether program delivery in different environments is associated with differential outcomes.

Despite the promising results, the present study is not without limitations. This study should be interpreted as exploratory and pilot in nature for several reasons. First, and most importantly, participants were allocated to groups based on their preference rather than random assignment. This non-randomized, quasi-experimental design significantly reduces internal validity and the strength of causal conclusions, as pre-existing differences between groups (e.g., motivation, attitudes toward exercise, or baseline physical condition) may have influenced the observed outcomes. The participants in this study constitute a small convenience sample, consisting of 27 older women who attend social centers in a region of Portugal, which is not very representative of the target population, preventing the generalization of the results. The fact that only older women participated can be considered a selection bias and may impact the validity of the results, as well as the generalization of the results to male individuals. At the same time, the low exercise frequency (one session/week) is below ACSM recommendations [35] and may limit the generalizability of the findings to higher-dose programs. The lack of control over external variables (e.g., stress, motivation, emotional state, sensory engagement, diet, hydration, and/or changes in participants’ medication) may have influenced the results obtained. It was also not possible to ensure that the assessments were carried out according to the recommended protocol, namely in the morning and on an empty stomach, due to institutional logistics and the characteristics of the participants themselves. Therefore, the results should be interpreted with caution. In addition, it was not possible to control the participation of older women in other physical activities outside the context of the study, which may have affected performance and results differently among participants. No long-term follow-up was conducted, so it is unknown whether the observed changes were maintained. Another methodological limitation relates to the age distribution of the groups: homogeneous stratification would have allowed for more balanced comparisons and stricter control of this potentially influential variable. The voluntary choice of the group to which the older adults could belong also compromises internal validity and introduces selection bias; on the other hand, it promotes self-determination, autonomy of choice and belonging to a group of their choice. Assessors were not blinded to group allocation, which may have introduced measurement bias. Finally, future studies should examine whether multisensory environments influence exercise responses through changes in perceived exertion, affective valence, stress regulation, enjoyment, or adherence.

## 5. Conclusions

This non-randomized exploratory pilot study suggests that a low-frequency, multicomponent physical exercise program delivered in community social centers is feasible and is associated with preliminary favorable changes in selected physical and psychosocial outcomes. The primary between-group analysis of change scores identified significant differences in body mass (Snoezelen vs. Exercise Room), Back Scratch (Snoezelen vs. Exercise Room) and Satisfaction with life (Snoezelen vs. Exercise Room and Exercise Room vs. Control). Secondary time-specific comparisons also showed group differences in bone mass, muscle mass, Back Scratch, 6-Minute Walk, and quality of life, but these findings are difficult to interpret causally because of baseline differences and the non-randomized design. The primary Δ findings do not demonstrate that the Snoezelen environment is superior to the conventional exercise room. Larger randomized controlled studies with baseline-adjusted analyses are needed to confirm whether the exercise environment contributes to differential outcomes.

## Figures and Tables

**Table 1:** Descriptive statistics of body composition results.

	Snoezelen Group			Exercise Room Group			Control Group			
	Mean ± Standard Deviation									
	Pre	Post	Δ	Pre	Post	Δ	Pre	Post	Δ	Pairwise Comparison (Δ Based)
**Body mass (kilograms)**	76.59 ± 10.53	74.90 ± 10.86	−1.68 ± 2.77	61.68 ± 15.75	60.72 ± 15.43	−0.95 ± 1.64	73.08 ± 13.29	74.40 ± 13.12	1.31 ± 0.89	H(2) = 9.516, *p* = 0.009 Pairwise comparison statistic: Snoezelen Group vs. Exercise Group =−3.012, p_adjusted = 0.008
**Fat mass (percentage)**	40.96 ± 4.31	39.48 ± 3.65	−1.47 ± 1.29	35.22 ± 9.21	35.01 ± 9.01	−0.21 ± 1.71	36.75 ± 5.90	36.90 ± 6.37	0.15 ± 3.11	a
**Bone mass (kilograms)**	2.27 ± 0.177	2.30 ± 0.22	0.03 ± 0.10	2.01 ± 0.27	1.95 ± 0.26	−0.05 ± 0.07	2.35 ± 0.27	2.33 ± 0.23	−0.01 ± 0.07	a
**Total body water (percentage)**	40.89 ± 2.60	41.87 ± 2.08	0.98 ± 0.92	43.47 ± 5.05	43.48 ± 4.79	0.01 ± 1.03	43.13 ± 4.00	43.53 ± 3.79	0.40 ± 2.92	a
**Muscle mass (kilograms)**	42.45 ± 3.49	43.07 ± 4.26	0.62 ± 1.81	37.51 ± 5.41	36.35 ± 4.98	−1.15 ± 1.97	43.38 ± 5.57	43.65 ± 4.07	0.26 ± 2.14	a
**Visceral fat (index from 1 to 59)**	13 ± 1.90	12 ± 3.65	−0.50 ± 0.70	11.14 ± 2.68	10.21 ± 2.76	−0.92 ± 1.20	12.26 ± 2.70	12.50 ± 3.01	0.23 ± 1.81	a

Note: H, Kruskal–Wallis omnibus test statistic; a, no differences detected; *p*, significance.

**Table 2:** Descriptive statistics of physical fitness results.

	Snoezelen Group			Exercise Room Group			Control Group			
	Mean ± Standard Deviation									
	Pre	Post	Δ	Pre	Post	Δ	Pre	Post	Δ	Pairwise Comparison (Δ Based)
**Chair Stand (repetitions)**	11.28 ± 3.68	13.50 ± 3.29	2.21 ± 1.84	9.85 ± 1.95	12.85 ± 1.21	3 ± 2.70	11 ± 1.41	11.83 ± 1.60	0.83 ± 1.32	a
**Arm Curl right (repetitions)**	16.57 ± 6.02	19.35 ± 5.42	2.78 ± 3.88	13.85 ± 5.30	18.42 ± 3.73	4.57 ± 3.25	17.50 ± 2.81	19.66 ± 3.32	2.16 ± 3.37	a
**Arm Curl left (repetitions)**	17.92 ± 4.56	20.14 ± 5.53	2.21 ± 4.62	13.71 ± 3.14	16.42 ± 2.37	2.71 ± 2.13	14.50 ± 1.87	18.66 ± 5.35	4.16 ± 4.49	a
**Chair Sit and Reach right (centimeters)**	3.28 ± 6.48	0 ± 0	−3.28 ± 6.48	0.42 ± 1.13	0 ± 0	−0.42 ± 1.13	2.50 ± 5.75	−0.66 ± 1.63	−3.16 ± 5.03	a
**Chair Sit and Reach left (centimeters)**	3.78 ± 7.65	0.85 ± 3.20	−2.92 ± 6.39	0.71 ± 1.88	0 ± 0	−0.71 ± 1.88	2.41 ± 8.63	−1.00 ± 2.44	−3.41 ± 7.45	a
**Timed Get-Up-and-Go (seconds)**	11.89 ± 5.58	11.03 ± 5.53	−0.85 ± 2	9.05 ± 2.32	8.87 ± 2.64	−0.17 ± 1.49	10.78 ± 3.48	10.84 ± 4.89	0.06 ± 2.77	a
**Back Scratch (centimeters)**	22.85 ± 9.33	28.42 ± 12.77	5.57 ± 8.72	17.28 ± 9.81	11.42 ± 8.61	−5.85 ± 8.64	33.16 ± 14.89	36.16 ± 21.85	3 ± 8.83	H(2) = 7.398, *p* = 0.025 Pairwise com-parison statistic: Snoezelen Group vs. Exercise Group = 2.719, p_adjusted = 0.020
**6-Minute Walk (meters)**	345.71 ± 33.67	322.85 ± 138.08	−22.85 ± 156.66	531.42 ± 59.84	577.14 ± 55.89	45.71 ± 42.76	353.33 ± 191.69	313.33 ± 250.97	−40 ± 177.08	a

Note: H, Kruskal–Wallis omnibus test statistic; a, no differences detected; *p*, significance.

**Table 3:** Descriptive statistics of the results of the Quality of Life and Satisfaction with Life.

	Snoezelen Group			Exercise Room Group			Control Group			
	Mean ± Standard Deviation									
	Pre	Post	Δ	Pre	Post	Δ	Pre	Post	Δ	Pairwise Comparison (Δ Based)
**Quality of life (score)**	28.28 ± 2.33	30.42 ± 3.05	2.14 ± 1.65	31.00 ± 3.16	35.42 ± 2.99	4.42 ± 1.81	27.66 ± 3.32	28.33 ± 2.16	0.66 ± 1.75	H(2) = 11.356, *p* = 0.003 Pairwise comparison statistic: Control Group vs. Exercise Room Group = 3.293, p_adjusted = 0.003
**Satisfaction with Life (score)**	4.52 ± 0.65	4.98 ± 0.74	0.45 ± 0.36	4.00 ± 0.47	4.02 ± 0.46	0.02 ± 0.33	4.56 ± 0.32	4.83 ± 0.58	0.26 ± 0.56	a

Note: H, Kruskal–Wallis (pairwise comparisons of group); a, no differences detected; *p*, significance.

**Table 4:** Difference between groups and moments for Body Composition results.

	Snoezelen Group		Exercise Room Group		Control Group		Pre												Post
	Median (Interquartile Range)						H	*df*	*p*	Between-Group Comparison at Pre	H	*df*	*p*	Between-Group Comparison at Post	H	*df*	*p*	Primary Analysis: Between-Group Δ	Supplementary Within-Group Analysis
	Pre	Post	Pre	Post	Pre	Post													
**Body mass (kilograms)**	75 (9.65)	72.6 (10.20)	60.10 (28.60)	58.80 (27.80)	70.85 (23.05)	72.20 (21.60)	4.576	2	*p* > 0.05	a	4.104	2	*p* > 0.05	a	9.516	2	*p* = 0.009	1 ≠ 2 (Post hoc = −3.012; p_adj = 0.008)	initial ≠ final (Snoezelen: Z = −1.988; p_adj = 0.047; r = −0.531 and Control Group: Z = 1.992; p_adj = 0.046; r = 0.813)
**Fat mass (percentage)**	41.40 (7.20)	40.20 (5.63)	33.20 (17.20)	33.40 (15.80)	36.00 (11.43)	36.90 (10.30)	2.463	2	*p* > 0.05	a	1.473	2	*p* > 0.05	a	2.465	2	*p* > 0.05	a	initial ≠ final (Snoezelen: Z = −3.077; p_adj = 0.002; r = 0.822)
**Bone mass (kilograms)**	2.30 (0.12)	2.25 (0.22)	2.10 (0.50)	2 (0.50)	2.35 (0.55)	2.30 (0.45)	5.470	2	*p* > 0.05	a	8.460	2	*p* = 0.015	1 ≠ 2 (Post hoc = 2.630; p_adj = 0.026) and 2≠3 (Post hoc = -2.492; p_adj = 0.038)	3.431	2	*p* > 0.05	a	
**Total body water (percentage)**	40.45 (4.40)	41.10 (3.42)	45.30 (10.40)	45 (9.90)	43.10 (7.20)	43.60 (6.05)	1.925	2	*p* > 0.05	a	1.014	2	*p* > 0.05	a	1.994	2	*p* > 0.05	a	initial ≠ final (Snoezelen: Z = 2.938; p_adj = 0.003; r = 0.785)
**Muscle mass (kilograms)**	42.50 (3.23)	42.80 (5.43)	38.10 (11.90)	37.70 (9.10)	43.65 (10.75)	42.70 (7.47)	4.487	2	*p* > 0.05	a	7.748	2	*p* = 0.021	1 ≠ 2 (Post hoc = 2.527; p_adj = 0.034)	4.114	2	*p* > 0.05	a	
**Visceral fat (index from 1 to 59)**	13.25 (3.25)	12.25 (2.13)	11.00 (4.00)	9.00 (5.00)	12.25 (4.57)	12 (3.75)	2.904	2	*p* > 0.05	a	4.425	2	*p* > 0.05	a	1.086	2	*p* > 0.05	a	initial ≠ final (Snoezelen: Z = −2.489; p_adj = 0.013; r = −0.665 and Exercise Room Group: Z = −2.041; p_adj = 0.041; r = 0.771)

Note: Values are presented as median (interquartile range). H, omnibus Kruskal–Wallis test statistic; df, degrees of freedom; p, unadjusted omnibus *p*-value; a, no differences detected; p_adj, Bonferroni-adjusted *p*-value for pairwise post hoc comparisons; Z, Wilcoxon signed-rank test statistic; r, Wilcoxon effect size; Pairwise post hoc comparisons were performed only following a significant omnibus Kruskal–Wallis test; 1 = Snoezelen Room Group, 2 = Exercise Room Group, 3 = Control Group.

**Table 5:** Difference between groups and moments for Physical Fitness results.

	Snoezelen Group		Exercise Room Group		Control Group		Pre											Post	
	Median (Interquartile Range)						*H*	*df*	*p*	Between-Group Comparison at Pre	*H*	*df*	*p*	Between-Group Comparison at Post	*H*	*df*	*p*	Primary Analysis: Between-Group Δ	Supplementary Within-Group Analysis
	Pre	Post	Pre	Post	Pre	Post													
**Chair Stand (repetitions)**	12.00 (6)	14.50 (5.50)	10.00 (2)	12.00 (2.00)	11.00 (2.50)	12.00 (3.25)	1.515	2	*p* > 0.05	a	2.095	2	*p* > 0.05	a	3.997	2	*p* > 0.05	a	initial ≠ final (Snoezelen: Z = 2.953; p_adj = 0.003; r = 0.789 and Exercise Room Group: Z = 2.207; p_adj = 0.027; r = 0.834)
**Arm Curl right (repetitions)**	16.50 (8)	18.00 (6.50)	14.00 (7)	18.00 (7.00)	16.00 (3.25)	20.00 (6.00)	3.025	2	*p* > 0.05	a	0.447	2	*p* > 0.05	a	1.790	2	*p* > 0.05	a	initial ≠ final (Snoezelen: Z = 2.080; p_adj = 0.038; r = 0.556 and Exercise Room Group: Z = 2.201; p_adj = 0.028; r = 0.832)
**Arm Curl left (repetitions)**	16.50 (5.75)	19.00 (7)	14.00 (3.00)	16.00 (5)	14.50 (3.50)	18.00 (7)	5.989	2	*p* > 0.05	a	2.520	2	*p* > 0.05	a	0.880	2	*p* > 0.05	a	initial ≠ final (Exercise Room Group: Z = 2.207; p_adj = 0.027; r = 0.834)
**Chair Sit & Reach right (centimeters)**	0 (5.25)	0 (0)	0 (0)	0 (0)	1.5 (6.50)	0 (1)	0.857	2	*p* > 0.05	a	3.500	2	*p* > 0.05	a	1.722	2	*p* > 0.05	a	a
**Chair Sit & Reach left (centimeters)**	0 (4.25)	0 (0)	0 (0)	0 (0)	0 (9.88)	0 (1.5)	0.569	2	*p* > 0.05	a	3.095	2	*p* > 0.05	a	0.508	2	*p* > 0.05	a	a
**Timed Get-Up-and-Go (seconds)**	11.05 (8.23)	9.30 (7.41)	7.62 (3.19)	8.17 (2.80)	10.66 (5.65)	9.17 (5.80)	1.492	2	*p* > 0.05	a	0.697	2	*p* > 0.05	a	0.901	2	*p* > 0.05	a	a
**Back Scratch (centimeters)**	24 (12.25)	28.00 (13.00)	19 (19)	16 (16)	36 (28.25)	36 (46.75)	3.891	2	*p* > 0.05	a	7.947	2	*p* = 0.031	1 ≠ 2 (Post hoc = 2.565; p_adj = 0.031) and 2 ≠ 3 (Post hoc = −2.396; p_adj = 0.050)	7.398	2	*p* = 0.025	1 ≠ 2 (Post hoc = 2.719; p_adj = 0.020)	initial ≠ final (Snoezelen: Z = 2.315; p_adj = 0.021; r = 0.619 and Exercise Room Group: Z = −2.043; p_adj = 0.041; r = 0.772)
**6-Minute Walk (meters)**	360 (40)	320 (220)	520 (80)	560 (80)	400 (410)	240 (510)	13.096	2	*p* = 0.001	1 ≠ 2 (Post hoc = −3.619; p_adj = 0.001)	9.015	2	*p* = 0.011	1 ≠ 2 (Post hoc = −2.871; p_adj = 0.012)	0.851	2	*p* > 0.05	a	initial ≠ final (Exercise Room Group: Z = 2.00; p_adj = 0.046; r = 0.756)

Note: Values are presented as median (interquartile range). H, omnibus Kruskal–Wallis test statistic; df, degrees of freedom; p, unadjusted omnibus *p*-value; a, no differences detected; p_adj, Bonferroni-adjusted *p*-value for pairwise post-hoc comparisons; Z, Wilcoxon signed-rank test statistic; r, Wilcoxon effect size; Pairwise post-hoc comparisons were performed only following a significant omnibus Kruskal–Wallis test; 1 = Snoezelen Room Group, 2 = Exercise Room Group, 3 = Control Group.

**Table 6:** Difference between groups and moments for Quality of Life and Satisfaction with Life results.

	Snoezelen Group		Exercise Room Group		Control Group		Pre										Post		
	Median (Interquartile Range)						*H*	*df*	*p*	Between-Group Comparison at Pre	*H*	*df*	*p*	Between-Group Comparison at Post	*H*	*df*	*p*	Primary Analysis: Between-Group Δ	Supplementary Within-Group Analysis
	Pre	Post	Pre	Post	Pre	Post													
**Quality of life (score)**	29.00 (3.50)	30.50 (3.50)	30 (5)	37 (6)	28 (6)	28.5 (3.75)	3.597	2	*p* > 0.05	a	12.659	2	*p* = 0.002	1 ≠ 2 (Post hoc = −2.676; p_adj = 0.022) e 2 ≠ 3 (Post hoc = 3.435; p_adj = 0.002)	4.452	2	*p* > 0.05	a	initial ≠ final (Snoezelen: Z = 2.958; p_adj = 0.003; r = 0.790 and Exercise Room Group: Z = 2.388; p_adj = 0.017; r = 0.902)
**Satisfaction with Life (score)**	4.60 (1)	4.80 (1.25)	4.20 (0.40)	4.20 (0.60)	4.60 (0.65)	4.80 (0.75)	4.072	2	*p* > 0.05	a	9.037	2	*p* = 0.011	1 ≠ 2 (Post hoc = 2.883; p_adj = 0.012)	11.356	2	*p* = 0.003	1 ≠ 2 (Post hoc = −2.442; p_adj = 0.046); 2 ≠ 3 (Post hoc = −3.293; p_adj = 0.003);	initial ≠ final (Snoezelen: Z = 2.965; p_adj = 0.003; r = 0.792)

Note: Values are presented as median (interquartile range). H, omnibus Kruskal–Wallis test statistic; df, degrees of freedom; p, unadjusted omnibus *p*-value; a, no differences detected; p_adj, Bonferroni-adjusted *p*-value for pairwise post-hoc comparisons; Z, Wilcoxon signed-rank test statistic; r, Wilcoxon effect size; Pairwise post-hoc comparisons were performed only following a significant omnibus Kruskal–Wallis test; 1 = Snoezelen Room Group, 2 = Exercise Room Group, 3 = Control Group.

## Data Availability

The data presented in this study are available on request from the corresponding author.

## Appendix A

### Visual Comparison of the Exercises Performed in the Snoezelen Room and in the Exercise Room Group

**Snoezelen Room Environment**		**Exercise Room Group Environment**
**Exercise:** Step up and down **Objective:** To work on lower limb strength and coordination		
	In the Snoezelen room, participants performed the exercises while watching a relaxing video and listening to calm music, which was designed to provide a multisensory environment. This type of multisensory stimulation was not present in the Exercise Room Group, where the exercises were performed in a conventional environment.	
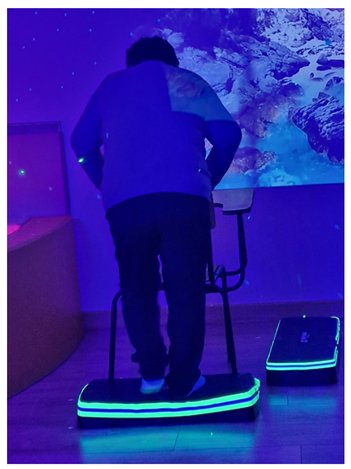
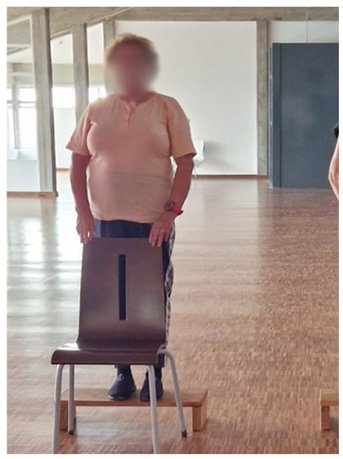
**Exercise:** Shoulder Press **Objective:** To work on upper body strength		
	In addition to soft music and relaxing videos, the Snoezelen room also included fiber optics/LED light strips, which provided ambient lighting and constantly touched the participants, as part of the Snoezelen room’s design features. These multisensory stimuli were not present in the Exercise Room Group.	
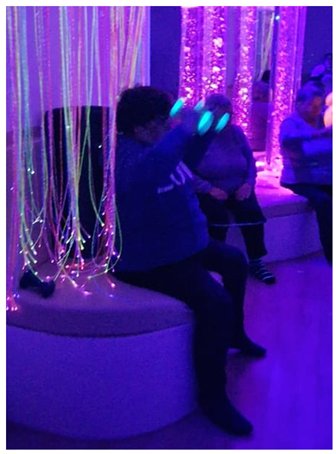
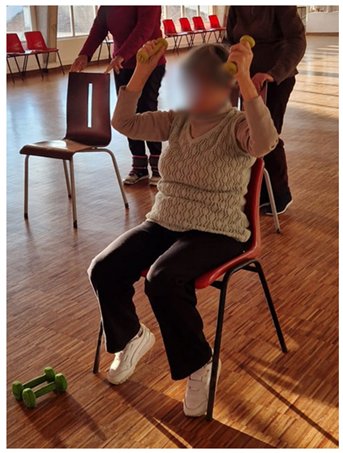
**Exercise:** Hip thrust **Objective:** To work on glute strength and improve core stability		
	In the Snoezelen room, in addition to calm music and relaxing videos, exercises were performed on a warm water bed. The warm water provided tactile and thermal input as part of the Snoezelen environment, and a column placed below the bed vibrated the water, which were characteristic of the Snoezelen room’s equipment. These elements, which were characteristic of the Snoezelen room, were not present in the Exercise Room Group.	
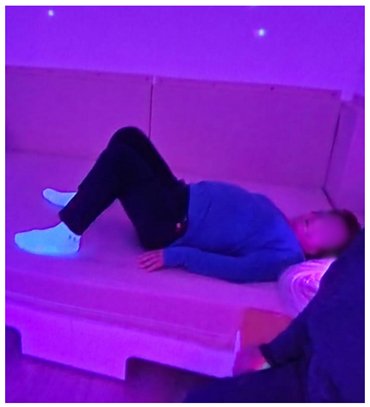
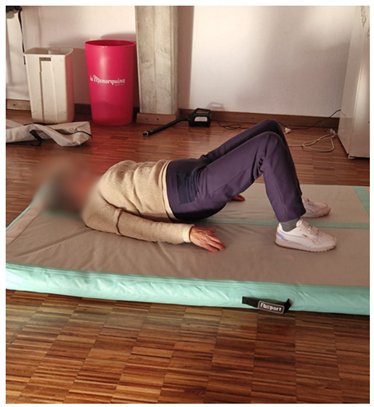
**Exercise:** Hip abduction with elastic band **Objective:** To work the hip abductors		
	In the Snoezelen room, in addition to calm music and relaxing video projections, participants performed exercises while leaning against a column of moving water. This equipment produced gentle vibrations and relaxing visual stimuli, which were part of the Snoezelen room’s multisensory equipment. This type of sensory stimulation was unique to the Snoezelen room.	
